# Standardized porcine robotic transversus abdominis release training: the SPORT model

**DOI:** 10.1007/s00464-025-12484-9

**Published:** 2025-12-15

**Authors:** Francesco Brucchi, Annabelle De Troyer, Maaike Vierstraete, Filip Muysoms

**Affiliations:** 1 University of Milan, Via Festa del Perdono, 7, Milan, Italy; 2 Department of General Surgery, Ziekenhuis Aan de Stroom (ZAS) Antwerp, Antwerp, Belgium; 3 Department of Plastic, Reconstructive and Aesthetic Surgery, ZAS Antwerp, Antwerp, Belgium; 4 Department of General Surgery, AZ Maria Middelares, Ghent, Belgium

**Keywords:** Robotic surgery, Transversus abdominis release, Abdominal wall reconstruction, Porcine model, Surgical training

## Abstract

**Background:**

Robotic platforms are increasingly adopted for complex abdominal wall reconstruction, yet transversus abdominis release (TAR) remains technically demanding with a steep learning curve. Structured training models are therefore essential to ensure safe implementation. Building on the experience of a previous model for inguinal hernia repair, the SPORT (Standardized POrcine Robotic Transversus abdominis release Training) model was developed as a stepwise, high-fidelity platform for robotic TAR.

**Methods:**

Ethical approval was obtained (EC2021/067) under the Belgian Animal Use Act. Crossbred pigs (Landrace × Large White, 14–16 weeks, ~ 50 kg) underwent open and laparoscopic dissections to assess abdominal wall anatomy. Based on these findings, a structured stepwise rTAR protocol was developed and tested on standard robotic platforms (da Vinci Xi, da Vinci X, Hugo RAS, Versius). This study does not include quantitative or qualitative performance data; rather, it provides a methodological description and validation of a standardized, reproducible training framework for robotic transversus abdominis release using a live porcine model.

**Results:**

The SPORT model reproduces key anatomical features of the human abdominal wall, including the arcuate line, the watershed zone at the costal margin, and the cranial extension of the transversus abdominis. A structured step-by-step description is provided, emphasizing critical landmarks, technical pitfalls, and system-specific docking maneuvers.

**Conclusions:**

The SPORT model offers a realistic and reproducible training framework for robotic TAR, allowing surgeons to practice both classical and modified techniques before performing them in human patients. By bridging the gap between synthetic or cadaveric training and live surgery, SPORT represents an essential component of structured curricula for robotic abdominal wall reconstruction.

**Supplementary Information:**

The online version contains supplementary material available at 10.1007/s00464-025-12484-9.

The implementation of robotic platforms in abdominal wall surgery has significantly expanded the range of procedures that can be performed with a minimally invasive approach. The European Hernia Society (EHS) has proposed a stepwise training pathway, emphasizing progressive acquisition of technical and procedural skills before clinical adoption [1]. This, and other similar, pathways recommends that surgeons begin with technology-oriented and simulator-based training, followed by hands-on practice on synthetic, cadaveric, or animal models, before progressing to low-complexity clinical cases [2, 3]. In hernia surgery, robotic transabdominal preperitoneal repair (rTAPP) has been proposed as the ideal index procedure for surgeons with previous laparoscopic experience, providing an opportunity to develop proficiency in dissection, mesh handling, suturing, and camera navigation. The SPIRIT model —a standardized porcine model for robotic inguinal hernia repair—has already demonstrated the value of live tissue models in facilitating skill acquisition in a realistic surgical environment [4].

Beyond index procedures, the true clinical impact of robotic technology in abdominal wall surgery lies in its application to complex hernias. Posterior component separation through transversus abdominis release (TAR), originally described by Novitsky et al., revolutionized the management of large and complex ventral hernias [5]. By enabling significant medialization of the fascial edges and placement of a large retromuscular mesh, TAR provides durable abdominal wall reconstruction while minimizing tension on the repair. When performed robotically (rTAR), the procedure combines the reconstructive benefits of TAR with the advantages of minimally invasive surgery, including reduced wound morbidity and shorter hospital stay [6, 7]. However, rTAR requires advanced technical expertise and a detailed understanding of abdominal wall anatomy, particularly in relation to the posterior rectus sheath, transversus abdominis muscle, and their associated neurovascular structures [8, 9]. Critical steps and landmarks for safe execution have been summarized by Grossi et al. in the “critical view of TAR,” underscoring the need for structured training before performing the procedure in clinical practice [10].

Cadaveric models provide high anatomical fidelity but are limited by availability, cost, and lack of physiological responses such as bleeding or tissue elasticity. Synthetic simulators are useful for early skills training but fail to replicate the complexity of live tissue dissection. In this context, the anesthetized porcine model offers an attractive compromise, combining anatomical similarity with the fidelity of live tissue handling [2]. Prior work has validated the porcine model for robotic inguinal hernia repair, but a standardized framework for rTAR training has not yet been described.

The present manuscript introduces the SPORT model (Standardized POrcine Robotic Transversus abdominis release Training), a structured, step-by-step training model for robotic TAR. The SPORT model aims to provide a reproducible and high-fidelity platform that enables surgeons to acquire the technical and anatomical expertise necessary for safe transition to clinical rTAR.

## Methods

All animal handling procedures complied with the guidelines of the Belgian Animal Use Act, and ethical approval was obtained under the reference EC2021/067. The pigs used in the study were crossbreeds between Landras and Large White, aged 14 to 16 weeks, with an approximate weight of 50 kg.

An anatomical study of the porcine ventral abdominal wall was first conducted through both open and laparoscopic dissections to assess its suitability for simulating robotic transversus abdominis release (rTAR) [11]. Particular attention was paid to the relationship between the rectus abdominis, posterior rectus sheath, transversus abdominis (TA), and peritoneum, with a focus on similarities and differences compared to human anatomy.

Based on these observations, a stepwise training protocol for robotic TAR was designed. The procedure was performed using standard robotic platforms (da Vinci Xi, da Vinci X, Hugo RAS, Versius), following a reproducible set-up and docking sequence. As shown in the video provided in the supplementary materials, the first side of the procedure was performed by the mentor, while the contralateral side was completed by the mentee, in order to reproduce a realistic training scenario.

The stepwise protocol was refined through approximately ten complete TR-400 rTAR training sessions performed by the senior author during the development of the SPORT model.

## Results

### Description of the porcine ventral abdominal wall

The porcine ventral abdominal wall closely resembles the human abdominal wall, with four main muscular layers: external oblique, internal oblique, transversus abdominis (TA), and rectus abdominis (RA). The RA is enclosed within a rectus sheath, formed by aponeurotic contributions of the lateral abdominal muscles, with a transition at the arcuate line similar to humans. The TA muscle is well developed, with a broad muscular portion that overlaps the RA in the upper abdomen. Its transition from muscular to aponeurotic fibers does not follow a straight semilunar line, but rather a bell-shaped curve, an important anatomical feature when simulating transversus abdominis release.

The posterior rectus sheath (PRS) is identifiable cranially, but below the arcuate line the rectus abdominis is covered only by transversalis fascia, comparable to human anatomy. The peritoneum is thicker in pigs than in humans, which can facilitate dissection but may reduce the realism of peritoneal handling. Vascular supply is dominated by the deep cranial epigastric pedicle, running along the posterior surface of the RA and branching similarly to human epigastric vessels, providing a useful landmark during dissection.

**Fig. 1:**
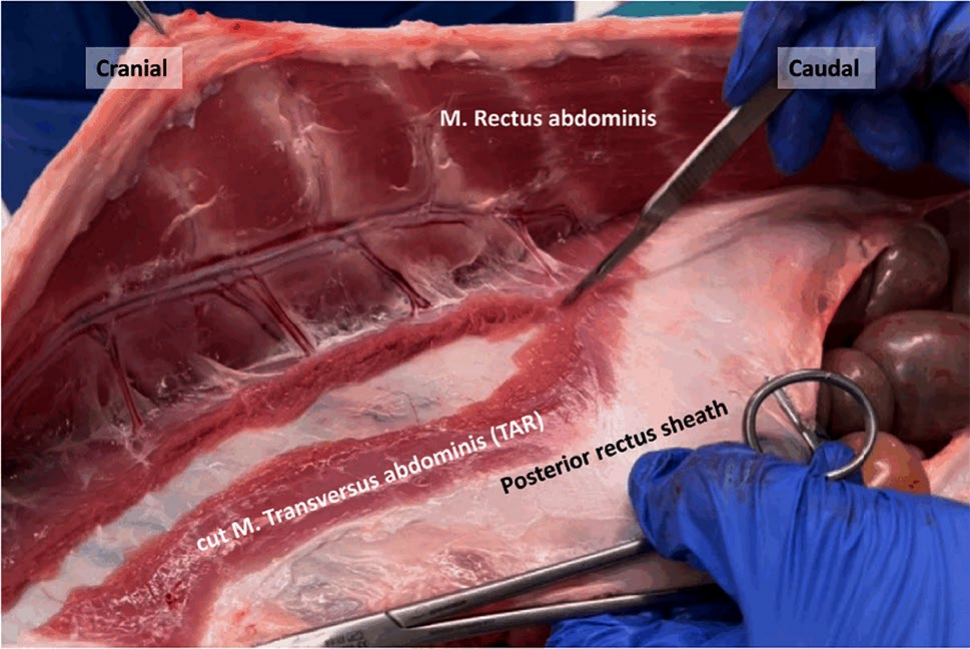
Transection of the M. Transversus abdominis resembling an open Transversus Abdominis Release (TAR) in a porcine model [12]

Overall, these anatomical characteristics confirm the suitability of the porcine model for training robotic TAR, enabling reproducible development of the retro-rectus plane, controlled incision of the TA, and lateral extension of the dissection. A more detailed description of the porcine ventral abdominal wall anatomy has been previously published and can serve as a reference for further anatomical insights (Fig. 1) [12].

## Description of the SPORT model

### Step 1 veress needle insufflation (Fig. 2)

**Fig. 2:**
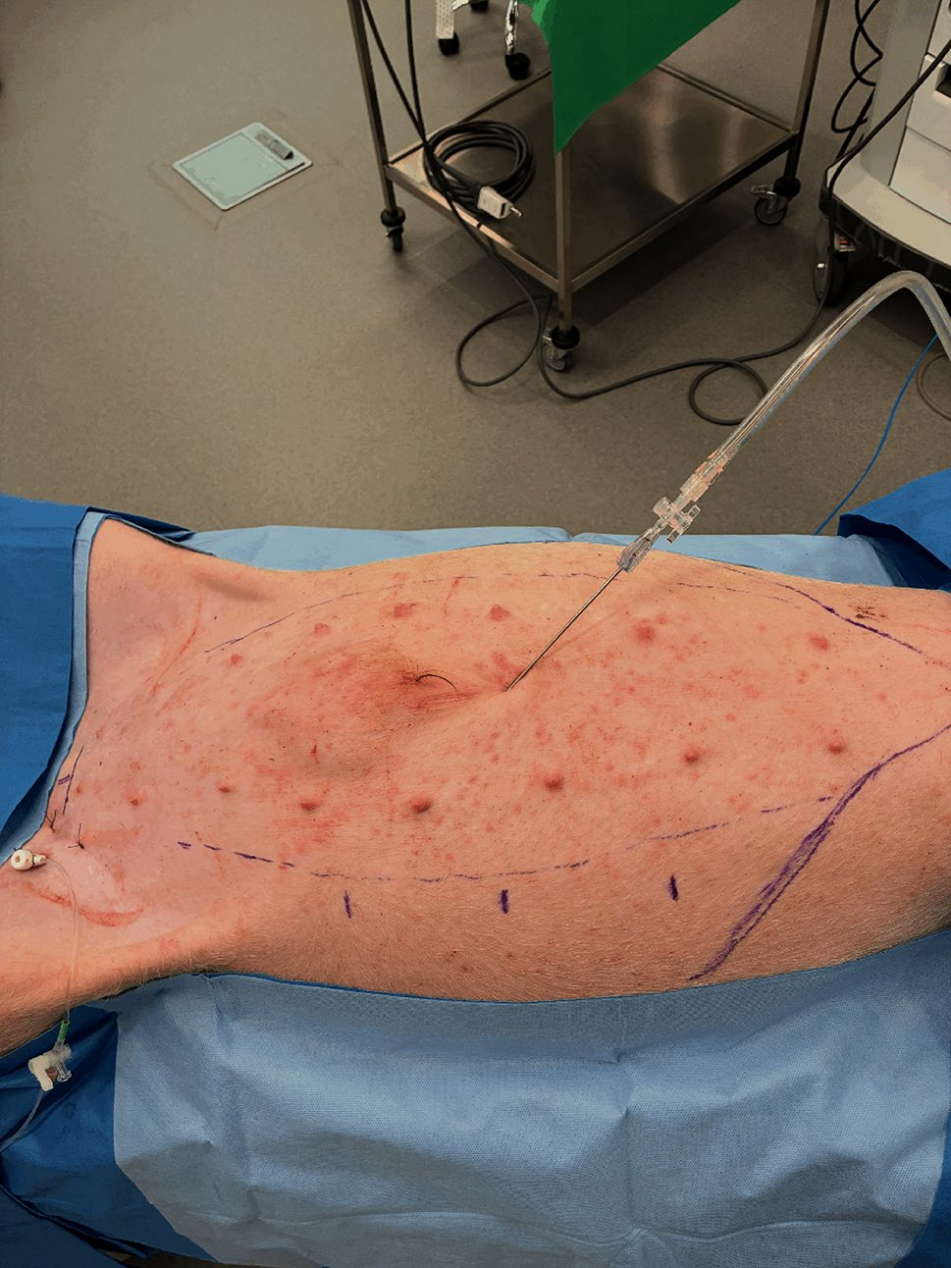
This figure shows the insertion point of the Veress needle, located above the penile bulge, together with the preoperative marking

**Fig. 3:**
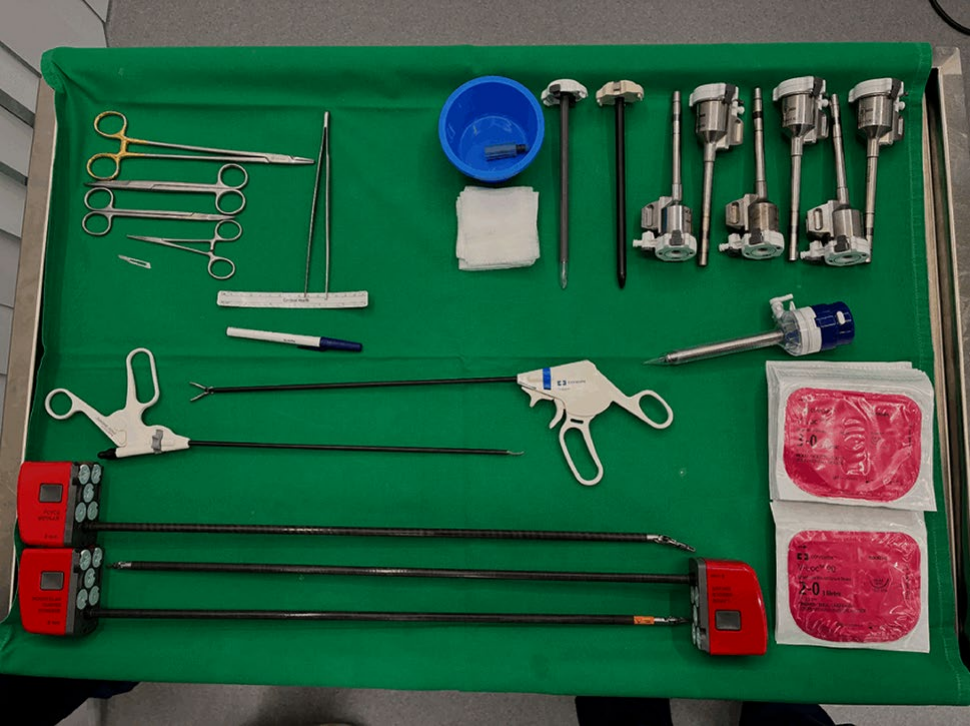
the complete set-up required to perform this procedure using the SPORT model

Establishing pneumoperitoneum in the porcine model requires particular caution, as pigs are highly sensitive to hemorrhage from solid organs and poorly tolerate prolonged high intra-abdominal pressures. Insufflation is best performed using a Veress needle introduced on the midline just above the penile bulge. In contrast to human surgery, where Palmer’s point in the left upper quadrant is often considered the safest entry site, this approach should be avoided in pigs because of the large splenic volume and the associated risk of life-threatening bleeding. For port placement, pneumoperitoneum is initially set at 15 mmHg and then reduced to 8 mmHg for the remainder of the procedure. Figure 3 illustrates the complete set-up required to perform this procedure using the SPORT model.

### Step 2 insertion of trocars

**Fig. 4:**
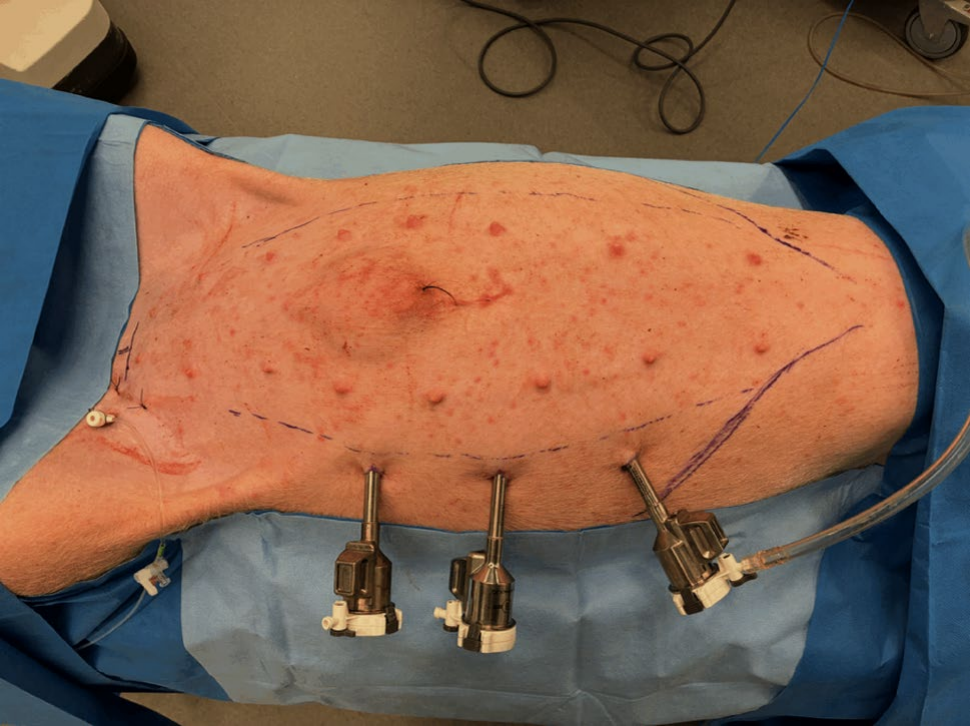
This image shows the first three 8-mm trocars positioned; in this case, the robotic system used was the da Vinci Xi

For robotic TAR in the porcine model, trocar placement follows a lateral approach. The first trocar for the scope is introduced on the flank, along the lateral abdominal wall, approximately at the level corresponding to the anterior axillary line in humans. Either the right or left side can be chosen as the starting point. Once the retro-rectus and TA planes have been fully developed on the initial side, three contralateral trocars are placed to allow completion of the dissection and mesh placement on the opposite side (Fig. 4).

For the da Vinci system, an 8 mm trocar is used for the camera, while the Hugo RAS and Versius systems require an 11 mm trocar. The working trocars are then inserted in a linear arrangement along the lateral abdominal wall, spaced to provide adequate triangulation and reach toward the midline. This configuration ensures optimal access for developing the retromuscular and lateral dissection planes necessary for TAR.

### Step 3 positioning of robotic arms

In the SPORT model, docking and positioning of the robotic system are performed in accordance with clinical practice for human rTAR. For the da Vinci Xi system, the “target anatomy” function allows the boom to rotate and be aligned toward the abdominal midline, facilitating bilateral access without the need to reposition the robot. In our setting, the target anatomy “Renal” was chosen to optimize alignment with the abdominal wall.

In contrast, the da Vinci X system has a fixed boom that cannot be rotated. Therefore, the cart must be positioned on the side of the porcine model corresponding to the flank where the initial dissection is performed. Once the first side has been completed, the system must be repositioned to the contralateral side in order to continue the dissection and complete the bilateral TAR.

For the Hugo RAS and Versius platforms, docking follows the same set-up guides as in human rTAR, with independent robotic arms placed laterally to the abdominal wall to optimize instrument reach and triangulation. Training on the SPORT model includes not only the dissection itself but also the docking maneuvers specific to each robotic system, ensuring realistic simulation of clinical conditions.

### Step 4 introduction of scope and instruments

For the SPORT model, the scope is introduced through the flank trocar placed on the side of the abdominal wall opposite to where the dissection is initiated. A 30° scope is recommended, as it provides optimal visualization of the retro-rectus and lateral dissection planes. Proper orientation of the camera is crucial to maintain adequate exposure of the posterior rectus sheath and the transversus abdominis muscle during the release.

As in clinical practice, correct introduction of the robotic instruments must always be performed under direct vision, given the absence of haptic feedback. This step is essential to avoid inadvertent injury during trocar entry and docking. The standard instrument set used for robotic TAR includes a monopolar curved scissors in the right hand and a fenestrated bipolar forceps in the left hand, although instrument choice may be adapted to the trainee’s preference and platform availability.

### Step 5 incision of the posterior rectus sheath and entry into the retro-rectus space

The dissection begins by identifying the medial border of the posterior rectus sheath. In many cases, this step requires gentle cleaning of the peritoneum and preperitoneal fat to delineate the linea alba and clearly expose the medial edge of the PRS. Once this border is visualized, a longitudinal incision is made, providing access to the retro-rectus plane.

After the incision, the retro-rectus plane is developed, separating the rectus muscle superiorly from the posterior sheath and peritoneum inferiorly. Dissection is extended caudally to the pubic symphysis and cranially toward the xiphoid, thereby creating an adequate working space for subsequent lateral extension and mesh placement. In the Madrid PCS (Posterior Component Separation), cranial dissection is usually limited to approximately 6–8 cm below the xiphoid. Beyond this point, the posterior rectus sheath is transitioned into the preperitoneal plane, allowing continuation of the dissection up to the xiphoid and preserving the anatomical insertion of the posterior rectus sheath on the costal cartilages [13].

Above the arcuate line, the posterior rectus sheath is well developed and easily dissected; caudal to this point, the rectus abdominis is covered only by transversalis fascia, as in humans. Recognizing this transition is critical, since it represents one of the anatomical variations that often confuses beginners during rTAR.

This step is fundamental for orienting the trainee to the correct anatomical planes and serves as the entry point for all subsequent phases of the transversus abdominis release.

### Step 6 identification and incision of the transversus abdominis muscle

Once the lateral edge of the PRS, also known as EIT Ambivium (MOE–MOI–TA junction; Musculus Obliquus Externus (MOE), the Musculus Obliquus Internus (MOI) and the Musculus Transversus Abdominis (TA)), is reached, the fibers of the transversus abdominis (TA) muscle become visible [9]. Their horizontal orientation differentiates them from the vertical fibers of the rectus abdominis and the oblique fibers of the internal oblique.

## Classical TAR

In the standard TAR technique, the TA is divided longitudinally to gain access into the lateral avascular plane between the muscle and the peritoneum. Dissection can proceed in two ways:

o Top-down: starting cranially, where the TA inserts onto the posterior rectus sheath, its fibers are divided and the endoabdominal fascia (TA fascia) is released laterally. The dissection is then continued upward across the costal margin onto the diaphragm and downward toward the retropubic space. Particular attention must be paid to the watershed zone at the costal margin, where the TA fascia may remain adherent to the peritoneum, and to the muscular fibers of the diaphragm, which may be mistaken for TA fibers and inadvertently injured.
o Bottom-up: starting caudally at the arcuate line, where the posterior sheath ends, the entry is straightforward and the dissection progresses cranially until the TA is divided and the fatty triangle and diaphragm are exposed.

For training purposes, we recommend performing a top-down dissection on one side and a bottom-up dissection on the contralateral side, thereby providing the trainee with a complete overview of both approaches and their respective anatomical challenges. Notably, the endoabdominal fascia behaves differently depending on the level: cranially, it remains adherent to the peritoneum, while caudally it stays attached to the transversus abdominis muscle. This variability requires careful recognition to avoid peritoneal breaches. It is important to note that this anatomical relationship is inverted in pigs, where the peritoneum is thicker cranially and thinner caudally, unlike in humans. This variability requires careful recognition to avoid peritoneal breaches.

## Madrid modification

In the Madrid posterior component separation, the TA muscle is preserved. Instead of incising the TA, the posterior rectus sheath itself is mobilized laterally and separated from the underlying TA fibers. This approach avoids division of the TA and potentially preserves neurovascular bundles (NVB) and reduces the risk of intercostal nerve or diaphragmatic injury near the costal margin. In the porcine model, however, the TA muscle is more developed cranially and extends more medially toward the linea alba compared with human anatomy. As a result, complete preservation of the TA fibers—one of the theoretical advantages of the Madrid modification—cannot always be achieved. Nevertheless, the principle of minimizing TA division and relying primarily on mobilization of the posterior rectus sheath is faithfully reproduced, making the model valuable for training in this alternative approach.

### Step 7 lateral dissection and plane development

After the transversus abdominis (TA) muscle has been incised, the dissection continues laterally within the plane between the TA and the underlying peritoneum. This step allows expansion of the retromuscular space and provides the medialization necessary for subsequent mesh placement.

Careful blunt and sharp dissection is performed to widen this avascular plane, proceeding laterally toward the abdominal sidewall. In the porcine model, the lateral limit of dissection is reached earlier than in humans due to differences in neurovascular bundle anatomy and the narrower abdominal cavity. Nevertheless, the essential features of the lateral release can be reproduced, offering realistic training in tissue handling, traction, and preservation of critical structures.

During this step, particular attention should be paid to preserve the integrity of the peritoneum, which is relatively thicker in pigs but still prone to tearing if excessive traction is applied. Proper development of this plane ensures sufficient overlap for mesh placement and reproduces the critical component of transversus abdominis release.

### Step 8 medialization and cranio-caudal extension of the dissection

Once the lateral plane has been created, the dissection is extended cranially and caudally to complete the retro-rectus and lateral release. Cranially, the dissection should reach the xiphoid process, where the muscular portion of the transversus abdominis comes close to the midline. Caudally, the dissection is carried down to the pubic symphysis. This cranio-caudal extension is essential to obtain a continuous retromuscular compartment that allows wide overlap of the mesh. Particular care must be taken to avoid injury to the epigastric pedicles, which in pigs are dominated by a prominent cranial branch running along the posterior surface of the rectus muscle.

This step reproduces the key reconstructive principle of TAR: the creation of a large, tension-free retromuscular space that permits safe placement of a wide mesh and durable reinforcement of the abdominal wall.

### Steps 5b–8b contralateral dissection

After completion of the first side, the same sequence of steps is repeated on the contralateral abdominal wall. This involves incision of the posterior rectus sheath (Step 5b), development of the retro-rectus plane, identification and incision of the transversus abdominis muscle (Step 6b), lateral dissection (Step 7b), and cranio-caudal extension with medialization (Step 8b).

For the da Vinci Xi platform, this transition is facilitated by simply rotating the boom to the opposite side, allowing continuous access without undocking. In contrast, for the da Vinci X, CMR Versius and the Hugo RAS systems, the robotic cart must be repositioned to the contralateral flank before proceeding with the dissection.

A specific difference during the contralateral dissection is that the previously placed trocars on the first side are encountered within the lateral plane. These trocars must be carefully freed from the abdominal wall layers during dissection, with the assistance of the bedside surgeon, to avoid inadvertent peritoneal tears or instrument entrapment.

This mirrored sequence ensures that both sides of the retro-rectus and transversus abdominis planes are connected, ultimately creating a continuous, large retromuscular compartment suitable for mesh reinforcement.

### Step 9 closure of the posterior layer and peritoneal defects

Before mesh insertion and suturing, a 12-mm laparoscopic trocar is placed in one of the unused lateral sites to allow the introduction of the mesh and sutures, as well as the retrieval of needles; this port is managed by the bedside assistant. Before mesh placement, the posterior rectus sheath and peritoneum are closed to reconstitute the posterior layer. Any openings created during trocar insertion into the lateral plane must also be carefully sutured under direct vision, in order to prevent direct contact between the mesh and the abdominal viscera. Closure is performed with a continuous barbed suture, paying particular attention to avoid gaps at the cranial and caudal limits of the dissection.

For optimal training, we recommend closing trocar-related peritoneal defects with interrupted 3–0 polyglactin (Vicryl™) sutures, while the posterior rectus sheath itself should be re-approximated with a continuous barbed suture (3–0). Particular care must be taken at the cranial and caudal limits of the dissection to avoid leaving gaps that could expose the mesh to the abdominal cavity.

### Step 10 plication of the linea alba and closure of the fascial defect

After restoration of the posterior layer, the linea alba and the medial fascial edges are approximated to close the midline defect. In the porcine model, this step reproduces the reconstructive principle of TAR by demonstrating medialization of the rectus abdominis muscles. A running or interrupted suture can be used to achieve tension-free closure, simulating the fascial reconstruction performed in human patients.

For optimal training, we recommend using a slowly absorbable barbed suture, size 0 or 1, with a minimum length of 30 cm. Whenever possible, we encourage the adoption of the Inan’s stitch (also known as the Geneva stitch), described as a running longitudinal slowly absorbable barbed suture (0 V-Loc™, 45 cm, Medtronic) using an imbricating suturing technique under low preperitoneal pressure (8 mmHg). This method creates an inward imbrication of the linea alba, minimizing the postoperative ridge that often occurs after more conventional suturing of the diastasis.

### Step 11 mesh placement (Fig. 5)

**Fig. 5:**
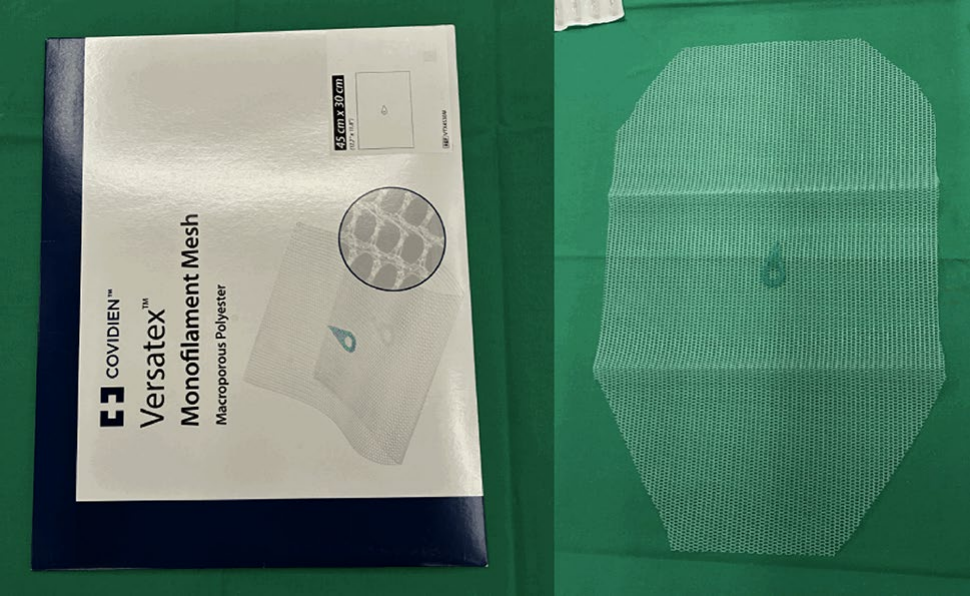
This image shows the mesh used in our case, a 30 × 45 cm Versatex™ (Covidien). On the right, the mesh is displayed after being tailored to fit the porcine anatomy

Once the posterior and anterior fascial layers have been restored, the mesh is introduced and deployed within the retromuscular compartment. In our case, a 45 × 30 cm macroporous polyester Versatex™ mesh (Covidien) was used after being appropriately tailored to fit the porcine anatomy. The mesh should cover the reconstructed midline and extend widely into the lateral dissected spaces to ensure sufficient overlap. In the porcine model, mesh size may need to be adapted to the different anatomy; however, the maneuver reproduces the principles of human abdominal wall reconstruction. Fixation can be performed with a limited number of sutures at key anchoring points, though in many cases retromuscular positioning ensures adequate stability.

### Step 12 desufflation and trocar removal

Before final desufflation, low-pressure inspection should be performed to confirm hemostasis and rule out concealed bleeding points. All needles and sutures are retrieved, and trocars are removed under direct vision. Fascial closure is recommended for ports ≥ 10 mm using slowly absorbable sutures.

### Video of the SPORT model

A video of the surgical technique and steps for the SPORT model is available as supplementary material to this manuscript.

## Discussion

The adoption of robotic technology for complex abdominal wall reconstruction, particularly for posterior component separation and transversus abdominis release (TAR), has created an urgent need for structured training pathways that ensure safe and effective implementation of these demanding procedures [1, 2]. While synthetic simulators provide an introduction to basic instrument handling, they lack the fidelity necessary to replicate the intricate anatomical planes involved in TAR. Cadaveric dissection offers superior anatomical realism but remains limited by availability, cost, and the absence of physiological tissue responses.

Robotic TAR is not routinely performed in the majority of centers, and its learning curve is long, technically demanding, and associated with significant complication rates when executed without sufficient experience. Because of this low procedural volume and the complexity of the operation, acquiring proficiency exclusively on human patients may require years, particularly in settings where exposure to complex abdominal wall reconstruction is limited. A structured and reproducible live tissue model therefore becomes essential, allowing trainees to familiarize themselves with the critical anatomical planes, high-risk steps, and technical pitfalls of the procedure before progressing to clinical practice. To address this, we developed the SPORT (Standardized POrcine Robotic Transversus abdominis release Training) model, a reproducible porcine-based platform for robotic TAR training. The porcine abdominal wall demonstrates high similarity with the human counterpart, including a well-developed posterior rectus sheath, a broad transversus abdominis muscle, and epigastric vascular pedicles that serve as reliable landmarks. Live tissue properties also provide realistic feedback for dissection, hemostasis, and suturing, which are critical skills for the safe execution of rTAR.

The porcine model reproduces all these anatomical scenarios with high fidelity: the broad muscular portion of the TA cranially (bell-shaped curve), the watershed zone at the costal margin, and the caudal arcuate line transition. Thus, SPORT provides trainees with the opportunity to practice both the classical TAR and the Madrid PCS, reinforcing correct identification of planes, awareness of anatomical variability, and safe handling of high-risk areas.

Importantly, the SPORT model reproduces both the classical TAR technique and contemporary modifications such as the Madrid PCS. Although the broader, more medial extension of the TA in the porcine model prevents complete muscle preservation, the SPORT model faithfully reproduces the principle of minimizing TA division and highlights the reconstructive endpoint shared by both approaches: the creation of a giant retromuscular space for wide mesh reinforcement.

One major advantage of the SPORT model is the high degree of standardization. In fact, it allows the establishment of error and critical error lists and supports the development of proficiency-based progression (PBP) metrics, as has been proposed in other robotic training pathways [14, 15]. Such metrics would enable objective assessment of performance and define thresholds that must be reached before advancing to clinical rTAR. In addition, the model trains not only surgical dissection but also system-specific docking maneuvers and intraoperative problem solving, such as arms conflict, trocar extraperitonealization and closure of peritoneal defects, which are critical to the safe performance of bilateral rTAR.

The SPORT model provides several educational advantages beyond anatomical fidelity. Its stepwise and reproducible set-up allows consistent replication of each operative phase, enabling the creation of standardized error lists and proficiency-based progression metrics that cannot be obtained with cadaveric or synthetic models. Unlike simulators, SPORT also trains system-specific docking, trocar placement, ergonomics, and the management of intraoperative challenges such as arm conflict or peritoneal defects. The model permits bilateral completion of the procedure and allows trainees to compare top-down and bottom-up releases within a single session. These features make SPORT not only anatomically realistic but also uniquely suited for integration into structured robotic abdominal wall curricula.

Despite these advantages, several limitations must be acknowledged. This study describes the SPORT model as a standardized porcine training platform for robotic TAR, but it does not present quantitative data on trainee performance, skill acquisition, or clinical outcomes. Its contribution is therefore primarily methodological and descriptive. Moreover, the model has not yet undergone external validation, and inter-rater reliability across different instructors was not assessed. Although the porcine anatomy closely resembles the human abdominal wall, species-specific differences—particularly in peritoneal thickness, cranial extension of the TA, and the absence of prior surgical scarring—may limit direct translational equivalence, and the model does not reproduce the complexity of incisional or recurrent hernias. In addition, biological variability among animals and the requirement for live tissue facilities may restrict widespread adoption, and we did not evaluate the logistical or cost implications associated with its implementation. Future research should therefore focus on prospective validation of the model, assessing how structured training on SPORT affects surgeon performance metrics, learning curves, operative time, and ultimately patient outcomes. Designing proficiency-based progression studies in which trainees are objectively assessed while using the SPORT model will be essential to define its role within robotic abdominal wall surgery curricula.

We believe the SPORT model represents a major step forward in robotic surgical training for complex abdominal wall reconstruction. By bridging the gap between synthetic or cadaveric training and live human surgery, it provides surgeons with a realistic and standardized platform to acquire both technical skills and anatomical knowledge. Ultimately, its integration into structured curricula could facilitate safer and more efficient adoption of robotic TAR in clinical practice, ensuring patients benefit from advanced reconstructive techniques performed with proficiency and confidence.

## Supplementary Information

Below is the link to the electronic supplementary material.

Supplementary file1 (M4V 51380 KB)
